# Abstracts and Gleanings

**Published:** 1883-03-20

**Authors:** 


### ABSTRACTS AND GLEANINGS.

**Koch's Theory of Tuberculosis.**—Dr. H. F. Formad, in a paper read before the Philadelphia County Medical Society, touching Koch's bacillus theory of tuberculosis, says:

My researches clearly show the following points:

1. The predisposition to tuberculosis in some men and animals, the so-called scrofulous habit, lies in the anatomy of the connective tissue of the individual, the peculiarity being a narrowness of the lymph-spaces, and their partial obliteration by cellular elements.

2. Only beings with such anomalous structure of connective tissue can have primary tuberculosis, and such animals invariably do become tuberculous from any injury resulting in inflammation, or from repeated injuries.

3. Scrofulous beings can have no other than a tuberculous inflammation, although it may remain local and harmless.

4. Non-scrofulous men or animals may acquire the predisposition to tuberculosis through malnutrition and confinement, the latter bringing on the above-mentioned anatomical peculiarities in the connective tissue.

5. No external etiological influences are necessary to cause tubercular disease other than those which ordinarily produce inflammation, and even scrofulous beings will not become tuberculous unless local inflammation is set up. No inflammation, no tuberculosis.

6. Non-scrofulous animals, so far as can be established now, may acquire tubercular disease through injuries of serous membranes,—viz: peritoneum, pleura, etc., and even here without any special virus whatsoever. Clinical observations on the post-mortem table show similar conditions and prove the same in man. (Koch's own experiments are also in favor of this proposition, as will be shown hereafter; but he has overlooked this.)

7. The bacilli, which it is the merit of Koch to have first proved to infest tissue affected by tubercular disease, are not necessary for its causation, even if a special organism exist and be really possessed of such property. The presence of bacilli (so far as our present research goes) is secondary, and appears to *condition* the complete destruction of the tissue already diseased and infested by them, and this destruction is in direct proportion to the quantity of the organisms, which thus regulate the prognosis. The tubercular tissue seems to serve merely as a nidus for the growth of the bacillus.

8. From the results of microscopic examination, from numerous observations upon the post-mortem table, and on clinical grounds, I have come to the conclusion that phthisis is not a specific infectious disease, but that the individuals suffering from tubercular disease are specific themselves originally, and form a special species of mankind, the "scrofulous."

9. Scrofulosis is a condition which may arise from malnutrition and seclusion in any being, and thus may be produced artificially.

It always depends upon the demonstrated anatomical changes in the connective tissue.

10. An analysis of Koch's experiments shows that he has not proved the parasitic nature of phthisis, or that there exists a special Bacillus tuberculosis; so that the infectiousness of tubercular disease is still *sub judice*.

In regard to infection he remarks: The natural history of tuberculosis is surely against the existence of a special poison such as now offered again by Koch. It is clearly proved that no infective agent is required to produce tuberculosis. It is possible that Koch's Bacillus tuberculosis in itself is capable of inducing the disease. There are at present no positive proofs either for or against it.

The evidence of those who have had a large experience with consumptive patients is in perfect opposition to the infective theory of phthisis. This, I think, is of more importance than experiments on the lower animals. The alleged fact that occasionally the healthy wife of a consumptive husband acquires phthisis (or the reverse), after prolonged cohabitation, can reasonably be explained by the presumption of an acquired scrofulosis from physical effects, misery of life, loss of sleep, etc.

Dr. Vincent Edwards, of the Brompton Hospital for Consumptives, testifies that during his seventeen years' experience and observations upon many thousand patients he has never observed a case of infection from person to person. None of his nurses ever contracted the disease.

The belief that milk or meat from tuberculous animals produces consumption when used as food, is also not warranted by scientific observation, nor is it based upon facts.

The natural history of tubercular disease and the laws of pathological physiology are against the presumption of a parasitic origin of phthisis.

We can certainly not have parasites more pernicious than the living cells of our own body prove to be in the case of tuberculosis. Our own cells (lymphoid cells) become dislodged from their natural location and move into other regions of the tissues, where they are not wanted, and where they do harm to the tissue they invade, and still more to themselves. They, however, continue to move through the body (as it seems, mainly by means of the perivascular spaces, the lymph-spaces proper being blocked up), everywhere leaving on their way small colonies of breeding cells which block up vital channels. These colonies of cells do not find enough nourishment in the new locations, and hence remain usually limited in size. Now the cells move closer together, forming nodes, to feed upon one another, and finally die and poison their host with the effete products of their dead bodies (cheesy degeneration.)

The ubiquitous bacteria, which (the bacillus included) linger around in countless numbers upon all surfaces without the least harm to a normal individual, easily penetrate a diseased tissue and make it a nidus for their growth. Young unripe cells created by morbid processes, frequently giant cells, which, under favorable conditions would have been transformed into a harmless connective tissue, from want of proper nutrition undergo retrograde change, and thus fall a prey to bacteria. While normal cells cannot be effected by bacteria (except by the anthrax bacillus, perhaps), morbid cells do not form (as I have myself seen) a good culture-medium for large crops of bacteria. Various kinds of bacteria (micrococci, rod-bacteria, bacilli, and vibrios or their spores) are present together in varying proportions everywhere. Different culture media favor however, the development of different kinds of bacteria: so all those new formations liable to cheesy change favor the predominant growth of *bacilli*. Here belong tubercle, leprosy, glanders, lupus, typhoid infiltrations, syphilis, swine-plague, and anthrax. Micrococci prefer the living blood and its white corpuscles as a medium for luxuriant development, if they succeed in getting across to it. The exanthemata and the ordinary kinds of septicæmia belong here. We cannot confirm, so far, that there is any difference between the micrococci of these last-named diseases, nor is it probable that a difference exists.

Koch has discovered that tubercle-tissue is always infested by bacilli, and this is correct; but this tubercle-tissue is not created on account of, or caused by, the bacilli. These organism, invade the tissue in question solely because it is a culture-medium favoring their predominant development.

As soon as tubercle-tissue undergoes complete cheesy degeneration and softening, the bacilli—Koch acknowledges this also—disappear from that locality nearly altogether, because no food is left and because the fat resulting in that process acts deleteriously upon them. This is also against the etiological relation of the bacilli with tuberculosis.

To consider, as Koch does, giant cells as mere special capsules of the bacilli, is a mistake not warranted by anything.

Koch further claims that the Bacillus tuberculosis differs from other bacilli morphologically, and in its behavior to staining fluids. We cannot confirm this. My assistant, Mr. Bodamer, and myself, after prolonged study with instruments as good as those of Koch, and after using all known methods of staining, have failed so far to see any special features in the bacillus in question which would make it distinct from other bacilli.

If Koch's bacillus even were possessed of distinct morphological features, it would not materially help to make it a specific organism.

Prof. Wood and myself made the observation that bacteria may acquire special morphological and physiological features in culture; excluding fully the possibility of Koch's "*Verunreinigungen*." Moreover, we have seen micrococci increase in size under certain conditions of culture. This is the more interesting, as Prof. Rothrock, of the University, made the suggestive observation that lower fungi or algæ, under culture, perhaps from pathological conditions of their own, may undergo decided, perhaps permanent, modification in their anatomy.

Whether or not the Bacillus tuberculosis stands in any causative relation at all with tuberculosis, only future investigations will show.

**Meniere's Disease.**—The following case from the British Medical Journal well illustrates the necessity of caution on the part of the public in forming opinions on matters relating to medicine: On the 21st of last October a court of inquiry was held to inquire into a charge of drunkenness preferred against a sub-constable. He had been seen to stagger and reel while on duty. He was taken to the barracks, where, in a short time, the transient attack of giddiness having passed away, he seemed, as he really was, perfectly sober. He was seen two hours afterwards by Dr. John Ringwood, when he exhibited well-marked symptoms of Meniere's disease; noise and hissing in his left ear, numbness behind the ears and down the left arm, depression, occasional vomiting, giddiness, objects going to the left side, the drum of the ear inflamed, and the left Eustachian tube plugged. Improvement followed inflation with the Eusfachian catheter.—*Med. and Surg. Reporter.*

**Electricity—Paralysis, Sciatica, Intercostal Neuralgia, Chronic Rheumatism.**—H. Mallory, M. D., in Medical Brief, says: I presume there are very few physicians, at this day and age, who question the utility of electricity as a remedial agent when directed by a skillful physician. But the high price of electric batteries, and their liability to get out of order, have caused many physicians to dispense with their use altogether. During the thirty-three years I have practiced medicine in Hamilton, I have bought half a dozen batteries, and have been not a little vexed when I had occasion to use my battery to find it would not work. It is to suggest an instrument that is free from these objections as well as to comply with personal and written requests of a large number of the medical profession that I write this article, and give to the profession my experience with the Electric Brush Battery. This is a regular Faradic battery, mounted on a metallic hair brush, and gives a current powerful enough for ordinary purposes, while at the same time it can be regulated to suit the most sensitive or delicate child. I have now been using this Electric Brush Battery for ten months, and have obtained such good results from its use that I have not found it necessary to use any other, and for the benefit of the medical profession I will give a few of the many cases I have treated with this battery.

The first case was that of myself, and its history is full of interest. During the month of February, 1881, I accidentally fell from a step-ladder, causing a severe concussion of the brain, followed almost immediately by paralysis of the right arm and leg, which kept me in bed for three months, during which time I was under the treatment of Dr. Daniel Millikin, of this place. At the end of this time I was advised by him to consult Dr. William Carson, of Cincinnati, who diagnosed my case and discovered a lesion or irritation of the right corpus striatum.

I remained under Dr. Carson's treatment until the 1st of July, and then, at his suggestion, went to the sea shore, where I took daily ocean baths until the second week in September. I returned home greatly improved in health but not cured. I now resumed the use of Electricity, having, by the direction of Drs. Millikin and

Carson,—from both of whom I derived much benefit—used it before I went to the sea-shore.

I now determined to give the Electric Brush Battery a trial, and commenced using it by applying it to the spinal column, the head, arm and leg, moistening the skin over the parts with water before each application. The effect was most gratifying, and I improved rapidly, and in less than two weeks I was free from the muscular twitchings in my legs and feet, which had existed since the date of my injury. I also recovered the use of my right hand, and was able to hold my pen and write—a thing I had not done for eight months. I soon regained strength to walk and attend to my practice, and while I have not entirely dispensed with the use of the battery (still using it two or three times a week) I am certainly as well as I ever was.

[Dr. Mallory goes on to describe five other cases, which we have not space to publish—one of sciatica, one of intercostal neuralgia, two of rheumatism, and one of facial neuralgia.]

**Ophthalmic Aphorisms**.—Dr. J. J. Chisholm, of Baltimore, gives the following valuable aphorisms in a report presented to the Maryland State Medical Society at its last session:

1st Aphorism.—Do not blister. In forty-nine applications out of fifty, as I find it used by physicians at large, it is an additional and useless torture to the eye diseases from which the patient is already suffering.

2d Aphorism.—Do not use nitrate of silver. As constantly prescribed by general practitioners, it is not beneficial in one case out of one hundred, and therefore is a very painful infliction to the ninety-nine who would have been so much better off without it.

3rd Aphorism.—Do not prescribe sugar of lead. In every case zinc, tannin or alum is better, and then there is no fear of having insoluble deposits incorporating themselves with the exposed surface of corneal ulcers.

4th Aphorism.—Always use weak solutions of the mineral and vegetable astringents in the treatment of eye inflammations which attack the mucus surfaces,and restrict their application to conjunctival diseases exclusively. One grain of alum, sulphate or chloride of zinc, sulphate of copper or nitrate of silver, in an ounce of water, will in the majority of cases of conjunctival diseases, do much more good and give much less uneasiness than the very painful five and ten grain solutions which are so often injuriously prescribed by physicians.

5th Aphorism.—Solution of the sulphate of atropia, from one to four grains to the ounce of rose water, is an essential eye-drop in the treatment of acute iritis, to break up newly formed adhesions. One drop of atropia solution in an inflamed eye is a most valuable means of establishing the diagnosis, whether iritic complications exist or not, and should be used in most cases of eye inflammation to find out whether there are any adhesions of the pupil to the lens.

6th Aphorism.—Eserine in solution of one grain to the ounce of water is the remedy for purely corneal lesions.

7TH APHORISM.—When physicians are in doubt as to the character of an eye disease, they should seek a consultation from specialists who are more familiar with the eye diseases than general practitioners can possibly be. Such timely aid often saves the patient a lifetime of trouble.

If physicians would commit to memory and keep at their finger ends, and ready for use, these simple aphorisms, the amount of mental and bodily suffering which they will prevent in their eye patients is beyond calculation. While all good rules have necessarily many exceptions, they may safely follow their simple guidance.—*Ohio Med. Journal.*

**Tracheotomy.**—Tracheotomy comprehends three steps: First, the opening of the air-tube; second, the introduction of a tracheal canal to keep patulous the opening; third, the subsequent dressings. Let us examine each one of these steps.

From the point of view of the opening to be made, the various operative procedures which are recommended may be arranged in several distinct classes. This division is based on the relative rapidity with which the operation is performed, or on the place where the opening is to be made. The question of place comprises the choice between laryngotomy, tracheotomy and crico-tracheotomy; that of relative rapidity includes the rapid, slow and mixed methods.

The rapid methods are the most seductive. To penetrate the trachea at once and in a few seconds to place a canula in the respiratory passages seems to be the method the most applicable in such cases, since it removes with extreme rapidity the obstacles which oppose respiration, and does away with that painful and difficult period of immobility demanded by the other procedure.

There are three methods of rapid tracheotomy—the one originated with my regretted master, Chassaignac, the other with Bourdillat, the third with my colleague and friend, St. Germain.

Chassaignac seized the cricoid cartilage with tenaculum furnished with a groove, serving to guide a bistoury with which he cut at one sweep the three first rings of the trachea; then this same tenaculum, after a modification which Isambert gave it, was made to open and serve as a dilator, in order to introduce the tracheal canula.

Bourdillat makes two stages of the operation: the first comprises the incision of the tissues down to the trachea; the second, the incision of this latter.

St. Germain proceeds in a somewhat different manner, and his method has been described by my former interne, Dubar, who has put it into practice in my service: The trachea is opened as you would open an abscess, the left hand grasping the neck makes the trachea prominent, and with a single thrust of the bistoury the air-passages are entered. A bistoury specially constructed enables you to limit the depth of the incision on the one part, and on the other enables you to hear the whiz of the air which escapes from the trachea at the moment of the incision; then, the incision being made, with a dilator you place the canula in the trachea.

I do not know what future is reserved for these rapid methods, which, indeed, have always seemed to me of the most simple kind. At the same time, used to the slow processes, I prefer them, while still recognizing the advantages of the quicker methods. Nevertheless, these advantages are at a discount when the operator fails, as he often will, speedily to introduce the canula; and this, because he attempts to do it through a wound which includes in the same extent the skin, subjacent tissues, and trachea.—*N. Y. Medical Record.*

**New Treatment for Purulent Conjunctivitis.**—The treatment Mr. H. L. Ferguson has been using at the Dublin Infirmary for many months past, consists in constant cleansing with an iced 4 per cent. solution of boracic acid, and when the more acute stage is over, touching the conjunctiva with a ten grain solution of nitrate of silver subsequently neutralized with a solution of salt.

The resnlts with this treatment were, on the whole, satisfactory, his observations coinciding with those of Mr. Simon Snell, published in the Ophthalmic Review for October; but convalescence was slow.

It occurred to him that if the boracic acid were applied to the conjunctiva in a finely powdered state its action would be quicker and more certain, and its application in this way seemed to be free from the objections to iodoform.

Briefly stated, the results of his observations have been: 1. That the application of the finely powdered boracic acid to a discharging conjunctiva checks the discharge completely for a period varying from two to twelve hours, and in the milder cases the first application is sufficient to stop the discharge altogether. 2. When the discharge reappears it is usually less in amount and more watery in characrer, and a very few applications of the powder stop it entirely. 3. The conjunctiva is then red and succulent but dry, and if touched two or three times with a solution of nitrate of silver it rapidly returns to its normal state.—*Eclectic Med. Jour.*

**Coffee as an Antidote to Alcoholism.**—By F. P. Novaes, Rio de Janeiro. The habitual use of coffee (*Caffea Arabica*) has been considered by some writers to be antidotal to alcoholism, and some of them apparently have no doubts upon the subject.

One of the gravest questions discussed in the Congress of Geneva was that of alcoholism, the means of fighting this terrible scourge, which from day to day makes such frightful progress in Europe, particularly in Switzerland.

His excellency, the Baron of Theresopolis, vice-director of the faculty of medicine of Rio de Janeiro, in some remarks made during the discussion of this topic, interested his audience and indicated the means he believed most efficacious to oppose the inroads of alcoholism. He produced statistics showing that the number of drunkards in a country is in inverse ratio to the amount of coffee consumed.

"In Brazil," he said, "where great quantities of coffee are used, and where all the inhabitants take it many times a day, alcoholism is completely unknown. It appears that the immigrants arriving in our country with this terrible passion for alcohol, contract little by little the habits of our people, imitating a fondness for drinking coffee, and their aversion for liquors. The children of these immigrants, brought up with coffee from their tender age, never contract the fatal habits of their parents.

"We can, therefore, conclude that the more coffee we take the less desire for alcohol we have. But to obtain such a result it is necessary that the coffee should be of superior quality, such as that from Brazil.

"Send us your emigrants," said the Baron; "we have work for them, and they will live under the protection of our government. In turn we will send you our coffee, which is the best remedy for such a trouble as this which you consider incurable."

His excellency may have exaggerated in this estimation of the effects of coffee, but we do not doubt that its use is an excellent antidote to alcoholism. The number of *cafes* in the large cities of Brazil, where hundreds of persons, from the highest down to the lowest classes of people, go in to take a cup of that delicious bevercge, which none but Brazilians know how to make properly, is enormous, whilst drinking saloons or bars are very few, and their patrons fewer still, in cousequence of which a public drunkard is a rare person to be seen.—*Phila. Med. Times.*

**Infantile Convulsions.**—The adopted and regular treatment of M. Jules Simon, of the Hopital des Enfants Malades, for infantile convulsions is as follows: On arrival the first thing he orders is an injection of salt and water, salad oil, or glycerine, or honey, which he administers himself, as he has too often observed that the parents or the nurse have already lost their wits. If the teeth can be opened sufficiently a vomitive is given which clears the stomach of any food that could not be digested—the most frequent cause of convulsions. However, the attack continues but soon ceases on applying a handkerchief, on which a few drops of chloroform are poured, to the mouth, which the child inhales largely. If the convulsions reappear the anæsthetic is renewed, and the child is placed in a mustard bath for a few minutes and then wiped dry and placed on his bed properly wrapped. Chloroform might be again administered if, after an interval, the child was seized again, and before leaving the nurse M. Simon prescribes a four ounce potion containing sixteen grains of bromide of potassium, one grain of musk, and a proportional preparation of opium, for he does not believe that the brain is congested in these attacks, it is rather excited, and the opium acts as a sedative. A teaspoonful of the mixture is given several times a day. On the following days the child is generally restless and irritable and ready to be attacked again, but a small blister about an inch square is applied to the back of the neck and left on about three hours, when it is replaced by a poultice of linseed meal, and gives most satisfactory results. M. Simon, in terminating, says "such is the treatment that I have instituted in my practice of every day."—*Med. Press.*

**Vertigo de Meniere.**—The patients afflicted with this disease are able to tell their own story, because they do not become completely unconscious. In the moment of the attack they hear a noise like that of an engine, and then they fall down forward, as if struck by a superior force, the latter being often so strong as to cause bruises of the nose or loss of teeth. After a while they will rise again and begin to vomit; they fall into a stupor, which lessens by degrees. After one or two weeks the attack will be repeated with the same phenomena. In a certain number of cases, the disease appears in the above described manner; the patients being well the rest of the time. But in a good many cases a permanent vertigo exists, with constant noises in the ear like that of a drum or a whistle. When the noise is aggravated, the attack will follow. M. Charcot has at the present time a patient who has been in bed for the last five years, and who avoids the least movement, which latter produces a feeling as if she would be raised in the bed very quick and then be lowered just as quickly. The noise may be due to an accumulation of cerumen in the ear, in which case the removal removes the noise. But more often it is due to otitis, or to another affection in the inner ear. M. Charcot treats these cases by the use of quinine, and says that this is a sure remedy.—*Journ. de Medic. et Chirurg. Prat.*

**Carbolic Poison.**—The following deaths from carbolic acid are reported: A man who had his hair clipped off was painted with carbolic acid over two-thirds of the head for some disease. He complained immediately about pains and dizziness in the head, became unconscious after a few minutes, and died shortly afterward. Three girls were painted tor scabies all over the body with impure carbolic acid, and they became unconscious after a few minutes. Two carpenters had used carbolic acid against scabies; one of them cried suddenly, became intoxicated, and died after a few minutes; the other became unconscious, but he recovered. A child of fourteen months fell upon a bottle filled with a strong solution of carbolic acid; the bottle broke, and the child was covered with the acid. Death followed a few minutes afterward. A midwife applied a small compress soaked with carbolic acid to a suppurating spot on a little child, which died after two hours. Experiments on animals have proved that carbolic acid brought in contact with the bowels or peritoneum will produce shock and collapse.—*Chicago Med. Ex.*

**Therapeutics of Chloral Hydrate.**—In the course of an article on this subject, in the St. Louis Medical and Surgical Journal, Dr. Joseph E. Harris recommends chloral for seasickness, 15-20 grains every four hours ; for the nausea and vomiting of pregnancy, in inflammations, eruptive fevers, etc., especially when attended by adynamic symptoms ; in cholera and cholera morbus, in nearly all nervous disorders, in delirium tremens, in puerperal eclampsia, in infantile convulsions, in tetanus, in whooping cough and spasm of the glottis, and concludes with the statement that hydrate of chloral is a nervous sedative, carminative, hypnotic, an antispasmodic, antiseptic, antiphlogistic, and we may say anæsthetic ; but gives the following caution : It should be prescribed with the greatest caution, if at all, when there is ischæmia of the arterial system. It should not be prescribed, for instance, in pneumonitis, in which there is bound to be a great disturbance of the pulmonary circulation.—*Med. and Surg. Rep.*

**Alcohol Inhalations in Croupal Diphtheria.**—Dr. Melsheimer, (Medical and Surgical Reporter) in the treatment of croupal diphtheria says : Now, if there is anything in the practice of medicine that is better calculated to humble the pride of the physician than a case of this kind, I have thus far failed to make the discovery, and I am very thankful for it. But we are called on to do something, and in our doing so we but demonstrate the impotency of our remedies and exhibit our ignorance of the cause to which they are directed for its removal. To be as consistent as possible with our imperfect knowledge of this disease, I prescribed stimulants in combination with iron, quinine, and chlorate of potash every three hours. Topical applications of tincture of iron and glycerin every four hours. Ordered the atmosphere of the room to be kept constantly moist by means of a large wash-boiler of hot water, and in connection used inhalations of a five per cent. solution of carbolic acid every four hours, by means of a small antiseptic atomizer. This treatment was continued with variations in inhalation for some sixty hours without any improvement whatever; in fact, grew from bad to worse right along. There was a progressive increased difficulty of breathing, with spasms of the muscles of the glottis, that threatened a speedy death from asphyxia. Her countenance and extremities were purple. Pulse, 160, thready and irregular, recession of the sternum and intercostal spaces, with each inspiratory effort; she appeared to be beyond the relief of medicine, yet, notwithstanding this hopeless condition I practiced the doctrine that as long as there was life there was hope. I abandoned all former treatment, with the exception of moist air, and resorted to the inhalations of hot alcohol, knowing that it would not compromise her condition in the least, and might possibly be the means of warding off death for a short time, at least; the alcohol used was rated at 88 per cent., and maintained at a temperature of 150° in the medicated cup. It was inhaled at a temperature of 120°, and reached the lungs through the nozzle of the atomizer, with a strength of about forty per cent. During the inhalation, which lasted about fifteen minutes, the pulse lost its intermittent character, and became somewhat fuller, the breathing more moist and less diffiult. The paroxysms, that threatened speedy dissolution, were relieved, and a decided improvement for the better gave some hopes of recovery. The inhalations were kept up at regular intervals of two hours through the night, and by the following morning she presented every promise of returning health. The blueness of the face and extremities was disappearing, her breathing was much less difficult; she expectorated large quantities of flaky, membranous matter, had increased secretion from the kidneys, and improved appetite. To facilitate the expectoration a preparation of syr. senega, spts. nit., and sanguinarine, in sufficient doses for this purpose, was prescribed every three hours. This, in fact, embraces the only treatment, and with the exception of a slight aphonia yet remaining, she is restored to health. At no time throughout the disease did the thermometer register more than 100°, with a low blood pressure, as was indicated by the character of the pulse. Her urine was scant; the eliminations from the kidneys seemed to hold a direct relation to the difficulty of breathing; at her worst period the amount of albuminous matter was equal by bulk to the urine.

**Jaborandi in Jaundice.**—We recently had a most obstinate case of jaundice, in which the usual remedies proved unavailing. We finally prescribed 30-drop doses of fluid extract jaborandi, with a view of relieving the circulation of the presence of bile through the skin. The sweating was profuse and great relief was afforded. The liver gradually resumed its action, aided by cream-tartar, podophyllin, extract taraxacum, etc. We attribute the starting of the function of the liver entirely to the action of the jaborandi.—*The Southern Clinic.*

**A New Method of Embalming.**—Instead of taking a solution of chloride of zinc, the following mixture is recommended: Thymol, 5 parts; alcohol, 45 parts; glycerine, 3,160 parts, and water, 1,080 parts. The injections made with this solution have the advantage of not being offensive, the instruments are not destroyed by it, and the bodies will be conserved any length of time, being mummified without putrefaction.—*Lyon Medicale.*

**Collecting Bills.**—A writer in Peoria Medical Monthly says: Don't be afraid of losing practice by collecting closely, for it is one of the best ways of holding it. Your patient probably owes all the other doctors in town, and sends for you because he has paid you, and consequently feels sure that you will attend him. Attend to your own collecting; you cannot trust to agents. Finally, we will meet with some that no art can reach; those we had better turn over to nature, especially when they get sick.

**A New Narcotic.**—A drug, hailing from Queensland, has recently produced a considerable sensation in Australian medical circles, where it is at present only known by its quaint native name of "pitchery-bidgery." A sufficient dose of this substance produces absolute insensibility to pain. It has the peculiar property of enabling those who take it habitually to withstand fatigue and undergo physical exertion upon a low diet.—*N. Y. Medical Times.*

**Specific for Sore Throat.**—Dr. Robert N. Hormerzdji, of Chittenham, has come to the conclusion that salicylate of sodium is a specific for acute tonsillitis. He recommends about 15 grains every hour, until the most urgent symptoms are relieved, when the quantity is reduced to one half. He also uses a gargle of salicylate sodium, grs. 10, glycerine 1 oz., water 3 oz.—*Lancet & Clinic.*

